# Power, pitfalls, and potential for integrating computational literacy into undergraduate ecology courses

**DOI:** 10.1002/ece3.4363

**Published:** 2018-07-30

**Authors:** Kaitlin J. Farrell, Cayelan C. Carey

**Affiliations:** ^1^ Department of Biological Sciences Virginia Tech Blacksburg Virginia

**Keywords:** active learning, big data, computer programming, data science, education, R software, sensor data, teaching modules

## Abstract

Environmental research requires understanding nonlinear ecological dynamics that interact across multiple spatial and temporal scales. The analysis of long‐term and high‐frequency sensor data combined with simulation modeling enables interpretation of complex ecological phenomena, and the computational skills needed to conduct these analyses are increasingly being integrated into graduate student training programs in ecology. Despite its importance, however, computational literacy—that is, the ability to harness the power of computer technologies to accomplish tasks—is rarely taught in undergraduate ecology classrooms, representing a major gap in training students to tackle complex environmental challenges. Through our experience developing undergraduate curricula in long‐term and high‐frequency data analysis and simulation modeling for two environmental science pedagogical initiatives, Project EDDIE (Environmental Data‐Driven Inquiry and Exploration) and Macrosystems EDDIE, we have found that students often feel intimidated by computational tasks, which is compounded by the lack of familiarity with software (e.g., R) and the steep learning curves associated with script‐based analytical tools. The use of prepackaged, flexible modules that introduce programming as a mechanism to explore environmental datasets and teach inquiry‐based ecology, such as those developed for Project EDDIE and Macrosystems EDDIE, can significantly increase students’ experience and comfort levels with advanced computational tools. These types of modules in turn provide great potential for empowering students with the computational literacy needed to ask ecological questions and test hypotheses on their own. As continental‐scale sensor observatory networks rapidly expand the availability of long‐term and high‐frequency data, students with the skills to manipulate, visualize, and interpret such data will be well‐prepared for diverse careers in data science, and will help advance the future of open, reproducible science in ecology.

## 1 INTRODUCTION

Computational literacy, or the ability to harness the power of computer technologies to accomplish tasks and solve problems (sensu Sherin, 2011), is increasingly required to conduct ecological research. Within the discipline of ecology, computational literacy encompasses confidence and familiarity in using technological tools to answer ecological questions. These tools, which include both point‐and‐click spreadsheet and statistical programs (e.g., Microsoft Excel) and scripting language‐based programs (e.g., R software), enable researchers to manipulate and analyze large multivariate datasets as well as perform computationally‐intensive analyses and visualizations. The explosion of the use of these tools – especially open‐source R software – among ecologists during the past decade is transforming the way ecology is conducted (Michener & Jones, 2012).

While opportunities to gain computational literacy are increasing at the graduate student level (e.g., Read et al., 2016), there remains a major gap in providing training in these concepts to undergraduate ecology students. As it is challenging to teach computational literacy to students that likely have limited programming experience, it is not surprising that R‐based laboratory activities are not common in undergraduate ecology curricula. However, teaching computational literacy to intermediate and upper‐level undergraduates may be the ideal time to expose students to these skills to help them prepare for diverse future careers. Moreover, embedding prepackaged, flexible modules into undergraduate classrooms to teach inquiry‐based ecology, as is done through Project EDDIE (Environmental Data‐Driven Inquiry and Exploration, http://www.ProjectEDDIE.org; Carey & Gougis, 2017; Klug, Carey, Richardson, & Gougis, 2017; O'Reilly et al., 2017), Macrosystems EDDIE (http://www.MacrosystemsEDDIE.org), Data Carpentry (von Hardenberg et al., 2018), and other projects that integrate data science and ecology (Table 1) may provide a viable solution for overcoming the pitfalls of programming instruction while stimulating undergraduates to think about ecology in a predictive way.

**Table 1:** Examples of modular teaching resources developed to bring modeling and/or data science principles into undergraduate ecology classes

Tool Name	Website	Platform	Description
Project EDDIE (Environmental Data‐Driven Inquiry and Exploration)	http://www.ProjectEDDIE.org	Excel (9 modules), R (1 module)	Modules use long‐term and high‐frequency meteorological, water quality, terrestrial, and geological datasets to model environmental phenomena
Macrosystems EDDIE	http://www.MacrosystemsEDDIE.org	R	Modules use long‐term and high‐frequency meteorological and water quality datasets to model macrosystems ecology concepts
QUBES (Quantitative Undergraduate Biology Education and Synthesis)	https://qubeshub.org/	Excel, Python, R	A clearinghouse of resources developed by math and biology educators designed to teach students how to tackle complex biological problems
Data Carpentry Ecology Curriculum	http://www.datacarpentry.org/lessons/	Python, R	Modules use a long‐term dataset of small mammal surveys to teach data manipulation, analysis, and visualization
SERC (Science Education Resource Center at Carleton College) InTeGrate Curriculum	https://serc.carleton.edu/integrate/index.html	Excel, Python, Stella	A clearinghouse of resources developed to foster interdisciplinary systems thinking in undergraduate environmental science courses
NEON (National Ecological Observatory Network) Teaching Modules	http://www.neonscience.org/resources/teaching-modules	Excel, R	A clearinghouse for modules that use NEON long‐term and high‐frequency data.

Here, we share our experiences developing and teaching undergraduate curricula as part of Project EDDIE and Macrosystems EDDIE, two U.S. National Science Foundation (NSF)‐supported initiatives to integrate computational literacy into undergraduate ecology classrooms. Within Project EDDIE, modules focus on teaching students to manipulate and interpret long‐term and high‐frequency data through problem‐solving lessons in the environmental sciences (see O'Reilly et al., 2017 for full module descriptions). Each EDDIE module has a scaffolding structure with three or more data analysis or modeling activities that build from simple to more complex and are grounded in the pedagogy of the 5E learning cycle (engagement, exploration, explanation, elaboration, and evaluation; Bybee et al., 2006). This flexible format enables instructors to choose the activities that are most appropriate for their classroom, as some module activities can be completed in a 1‐hr lecture period, whereas the entire module could be taught during a 3‐hr laboratory session. This flexibility allows the EDDIE modules to be taught in classes across a range of student experience levels.

The Macrosystems EDDIE project builds on the pedagogical framework of Project EDDIE to develop an R‐based curriculum to teach students fundamental macrosystems topics, with a focus on understanding drivers and ecological responses that operate at multiple, interconnected spatial and temporal scales (sensu Heffernan et al., 2014). In contrast to the Project EDDIE modules, which are Microsoft Excel‐based except for the Lake Modeling module, all of the Macrosystems EDDIE modules developed to date use the R statistical environment to introduce students to basic programming skills while conducting whole‐ecosystem simulation modeling and data visualization. All teaching materials for the Macrosystems EDDIE modules are available at http://www.MacrosystemsEDDIE.org. In the Macrosystems EDDIE modules, a primary goal is to teach computational literacy and computer programming skills as a means to ask and answer complex questions in ecology.

Here, we assessed the efficacy of these three R‐based EDDIE modules (“Lake Modeling”, “Climate Change Effects on Lake Temperatures”, and “Cross‐Scale Emergence”) on student skills and comfort with different components of computational literacy in ecology classrooms from a range of institution types through the use of pre‐ and post‐module student questionnaires. Below, we share the insights we developed from working on the EDDIE initiatives to justify why computational literacy needs to be better integrated into undergraduate ecology courses (*Power*); examine challenges related to teaching computational skills to undergraduates, with associated solutions (*Pitfalls)*; and present data supporting the use of EDDIE modules for building the computational literacy of undergraduate ecology students (*Potential*).

## 2 POWER

Ecologists are increasingly collecting and analyzing large datasets of long‐term and high‐frequency sensor observations (Hampton et al., 2013; Weathers et al., 2013). As working with “big data” becomes more common in ecological research, introductory students need to become adept at using a range of computational tools to manipulate, analyze, and interpret such data (Durden, Luo, Alexander, Flanagan, & Grossmann, 2017). An important first step is mastering spreadsheet programs, but ultimately students need more advanced skills in data manipulation, analysis, and visualization that are simply not possible in “point and click” programs, and which require training in programming languages such as R, Python, C, and others. Environmental sensor networks including GLEON (the Global Lake Ecological Observatory Network), NEON (the National Ecological Observatory Network), and others now regularly collect datasets containing millions and even billions of observations, which spreadsheet software programs cannot easily handle. Moreover, dragging, selecting, or clicking on rows or columns in such large datasets for data manipulation is both time‐prohibitive and error‐prone.

There are many benefits for undergraduate ecology students learning basic programming skills. First, programming enables reproducible, “open science” (Hampton et al., 2015). Sharing annotated code through open repositories such as GitHub enables and accelerates collaboration and the communication of ideas in ways that are not possible with spreadsheet software (e.g., a large distributed team can simultaneously edit the same code with version control in GitHub). Ecology researchers are increasingly using and sharing programming scripts to ensure the reproducibility of their analyses as well as to check for errors; these same benefits apply to using code to teach undergraduate students ecology concepts and analyses in the classroom. Second, programming languages provide functionality that is not available in spreadsheet programs. This includes a common format for collaboration and teaching across different computer versions or operating systems that can be difficult with spreadsheet software (e.g., Microsoft Excel files have embedded functions, macros, and analysis toolkits that cannot easily be shared among users with different computer operating systems or software versions). Third, as noted above, programming languages can be used to easily manipulate large datasets that spreadsheet software with row and column limits cannot handle. Fourth, several programming languages are open‐source, including R and Python, which enable undergraduates at all schools to use them without the need for expensive software site licenses. As a result, open‐source software broadens participation and facilitates a diverse community of users. Users can both access and contribute to open, community‐based technical support through blogs, open lab notebooks, and question and answer sites for programming such as Stack Overflow (Hampton et al., 2015). In addition, open‐source software enables community‐based development of code, resulting in the creation of many tools that have been developed specifically for ecological applications (e.g., R packages that calculate species diversity metrics).

Undergraduate students represent the next generation of scientists and need the best training possible to prepare them to tackle complex ecological research questions. Graduate students increasingly need to be able to analyze high‐frequency datasets early on in their dissertation research (Read et al., 2016), and thus the lack of training in computational literacy skills at the undergraduate level could hinder progress in ecology graduate education. Given that a recent survey of undergraduate ecology students found that the majority of respondents expect that they will need quantitative, data management, and/or database skills for their future careers (Carey, Gougis, Klug, O'Reilly, & Richardson, 2015), it is clear that many students are interested in receiving greater computational literacy training. Importantly, programming is increasingly being incorporated in undergraduate curricula across many STEM (science, technology, engineering, and mathematics) fields (e.g., Lawson, Szajda, & Barnett, 2013, National Research Council 2009; Wright, Provost, Roecklein‐Canfield, & Bell, 2013), indicating that ecological instruction needs to evolve to keep pace with related disciplines.

## 3 PITFALLS

Despite the power associated with increasing computational literacy in ecology undergraduate education, a number of pitfalls can hamper efforts to integrate computer science approaches and tools into existing curricula. These pitfalls can be overcome using strategies we have found to be successful for teaching the EDDIE modules.

**Figure 1:**
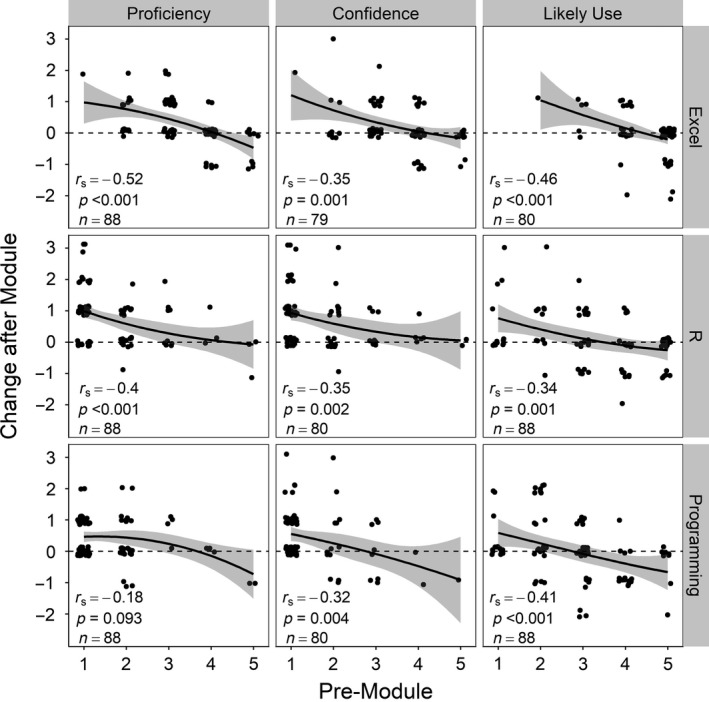
Changes in student post‐module self‐assessments of proficiency, confidence, and likely future use of quantitative tools relative to pre‐module responses. Students with the lowest self‐reported proficiency, confidence, and likely future use of a tool prior to completing a module exhibited the largest gains. Responses were on a Likert scale from 1 (low) to 5 (high; Appendix for methodological details). Points represent individual students and are jittered to show relative frequency of responses. Horizontal dashed lines represent no change in self‐assessment; positive values indicate increased proficiency, confidence, or likelihood of future use. Correlation coefficients (Spearman's rho), *p*‐values, and sample size (*n*) are shown for each panel. Solid lines show Loess smoothed fits, with 95% confidence intervals shaded in gray

First, in our experience developing and teaching EDDIE modules, we have found that intimidation by computational tools and the steep learning curve for programming is a key barrier to entry for many undergraduate ecology students. Self‐assessments by ecology students prior to using our modules have indicated that a majority of students recognize the importance of R statistical software and computer programming for future careers, but consider themselves to be minimally proficient and unconfident in using these tools, compared to intermediately proficient and confident with Microsoft Excel (Table 2, Appendix). This lack of proficiency and confidence renders students unlikely to attempt to learn R or other programming languages on their own (Baker, 2017). However, this discomfort can be overcome in part by having students modify and run existing, ready‐to‐use scripts and computer models, rather than developing scripts de novo. In addition, by breaking down complex activities into short, do‐able chunks of code, student skills are reinforced frequently as they work through module tasks. By designing ecology activities that assume no prior knowledge of programming (Baker, 2017) and use relevant, real‐world tasks (Valle & Berdanier, 2012), students are more likely to remain engaged in completing course activities, including EDDIE modules. Our experiences support this, as post‐module self‐assessments of EDDIE users indicated significantly higher perceptions of proficiency and confidence in using a suite of computational tools than in pre‐module self‐assessments (Table 2, Figure 1).

**Table 2:** Comparison of undergraduate student pre‐ and post‐module assessments of computational literacy based on paired, two‐sided Wilcoxon signed‐rank tests of students’ self‐reported proficiency, confidence, and likely future use of a computational tool (see Appendix for methodological details). Significant differences between pre‐ and post‐module responses are highlighted in bold (*α* = 0.05). Effect size was calculated as Z/√*n*

Metric	Test statistic	Two‐tailed *p* value	*n*	Pre‐module mean (±1 SE)	Post‐module mean (±1 SE)	Effect size
Microsoft Excel						
Proficiency	153.0	**0.001**	88	3.31 ± 0.09	3.61 ± 0.09	−0.37
Confidence	108.0	**0.010**	79	3.50 ± 0.09	3.76 ± 0.10	−0.29
Likely use	136.0	0.452	80	4.54 ± 0.07	4.56 ± 0.07	−0.08
R software						
Proficiency	43.0	**<0.001**	88	1.67 ± 0.09	2.44 ± 0.10	−0.68
Confidence	17.0	**<0.001**	80	1.77 ± 0.10	2.44 ± 0.12	−0.66
Likely use	390.0	0.408	88	3.27 ± 0.12	3.47 ± 0.13	−0.09
Programming						
Proficiency	95.0	**<0.001**	88	1.57 ± 0.08	1.93 ± 0.10	−0.51
Confidence	180.0	**<0.001**	80	1.56 ± 0.09	1.89 ± 0.11	−0.41
Likely use	502.5	0.864	88	2.74 ± 0.11	2.80 ± 0.12	−0.02

Second, while many consider current undergraduates to be “digital natives” (sensu Prensky, 2001), in that they have likely been exposed to computers from a relatively young age, individual experiences with computing can vary dramatically (Bennett & Maton, 2010; Wang, Myers, & Sundaram, 2013). Despite growing access to computers both in the classroom and at home, students have exhibited an increasing divide in their digital skills (Wang et al., 2013). In addition, day‐to‐day interactions with technological tools such as smartphones do not necessarily prepare students for advanced applications of computational tools, such as programming, and many students lack experience using computers as tools for scientific inquiry (Bennett & Maton, 2010; Wilensky, Brady, & Horn, 2014). Therefore, instructors may overestimate the technological skills of their students (Bennett & Maton, 2010 and references therein), creating a mismatch between the design of classroom activities and the capabilities of students. In response, instructors must recognize that “digital natives” are not a homogeneous group, and that some students may require additional technology instruction, especially when it comes to programming and other advanced activities (Wang et al., 2013). We have found that having students complete EDDIE activities with a partner can help equalize varying experience levels, as each partner brings unique computing experiences that can help the other complete module tasks. In addition, asking more advanced teams that finish the module early to assist their peers also equalizes disparities in classroom computational abilities. Finally, having instructors check in with students throughout the module to ask discussion questions ensures that students who may not be as technologically‐capable do not lag behind their peers in completing module activities. Repeated check‐ins by instructors and the use of module worksheets where students have to hand‐write answers to discussion questions also help keep the focus of the module on ecology concepts, not the computing tasks, by forcing students to interpret model outputs and connect those outputs to their prior ecological knowledge (sensu Sins, Savelsbergh, & van Joolingen, 2005) rather than simply run code without thinking critically about its utility and application.

Third, a lack of first‐hand experience troubleshooting software issues can exacerbate intimidation for students, hindering their motivation to increase their computational literacy. Many computer interfaces, including both personal computers and mobile devices, use “apps” that often hide source code and error messages from the end user. Thus, students have limited exposure to the code underlying software programs and often do not know how to respond when error messages occur during their first programming experiences, which may in turn undermine computational literacy (Blum, 2012). To overcome this discomfort, EDDIE modules include detailed instructions that walk students through each step of an activity. The inclusion of pictorial troubleshooting guides allows novice users to work through computer error messages or other hardware and software challenges on their own (e.g., how to identify the directory structure of files on their computer), without holding back more advanced users. In addition, we have found that having students complete modules using their own laptop computers makes troubleshooting software issues easier, as a student is likely more comfortable working with their own machine than a computer they have never used before. However, in instances when individual access to laptop computers may be limited, the use of a campus computer lab can help streamline access to tools, as well as ensure that all students are using the same up‐to‐date versions of necessary software.

The pitfalls described above can be exacerbated by a lack of instructor experience and comfort with advanced computational tools and the use of models (Louca & Zacharia, 2012). If faculty instructors do not actively use computer programming and computer‐based models, they are unlikely to teach their students these skills. Thus, a goal of Project EDDIE and Macrosystems EDDIE has been to develop training tools that are accessible to instructors from a range of institutions and with differing backgrounds and familiarity with the tools being taught. By incorporating basic training for instructors, we hope to empower instructors and build their confidence in using advanced computational tools, so that they in turn can share their knowledge with students. In addition, having undergraduate students observe their instructors using tools with which they may be initially uncomfortable can be a powerful catalyst for demonstrating that it is okay to struggle through unfamiliar territory, and that ultimately, everyone's knowledge increases by learning together.

## 4 POTENTIAL

Our work to date with Project EDDIE and Macrosystems EDDIE suggests that undergraduate students can substantially improve their computational literacy after short‐term (i.e., one laboratory period) engagement with activities designed to build such skills (Carey & Gougis, 2017; Klug et al., 2017). If ecology curricula begin to incorporate modules such as those developed by the EDDIE initiatives and other activities (e.g., Table 1) to increase computational literacy among undergraduates, we foresee tremendous potential in building student confidence and proficiency in the use of computational tools, and in turn, in their ability to do ecology in this era of big data.

Increasing computational literacy requires that students feel empowered, rather than intimidated, by advanced computational tools. When instructors foster a growth mindset (sensu Elliot, Dweck, & Yeager, 2017), students are able to view the challenges they encounter while working with computational tools as opportunities to build their skills rather than as criticism of their abilities. Indeed, qualitative student feedback in post‐module self‐assessments suggests that building student confidence in their ability to use such tools and troubleshoot errors lays the groundwork for ongoing growth in proficiency. For example, while one of our students initially expressed “frustration with my computer not working with the latest version of R,” they recognized that working through the challenges was “probably a good experience overall to show how powerful tools are sometimes frustrating to use but worth it for the output you can get once it works.”

Through guided activities that introduce computational tools, instructors can also empower undergraduate students with techniques to ask their own novel ecological questions and test hypotheses. Indeed, after completing an EDDIE module that used ecosystem modeling to explore the effects of climate change on lakes, some students felt empowered to continue working with the ecosystem model in R to conduct their own additional independent analyses, reflecting that they “enjoyed making our own simulations because of the freedom to be creative in how far we could push the model.” As with the analogy that teaching a person to fish provides longer‐lasting benefits than giving them a fish, learning computational approaches to test hypotheses provides longer‐term and lasting benefits by building students’ science self‐efficacy (sensu Ballen, Wieman, Salehi, Searle, & Zamudio, 2017) and helping them discover their own enduring, science‐related interests (Feinstein, Allen, & Jenkins, 2013).

Embedding hands‐on programming activities into undergraduate ecology curricula may also help advance the discipline as a whole toward being a more predictive science. Modeling‐based learning allows students to use simulations to test predictions about interconnected systems, leading to improvements in their understanding of scientific concepts (Louca & Zacharia, 2012). Learning about the process and purpose of modeling can also promote a more nuanced understanding of the scientific process (Schwarz & White, 2015). As a result of participation in the EDDIE modules, we have found that undergraduate students ask ecological questions in new and more powerful ways. For example, when asked in pre‐module assessments how they would estimate lake water temperatures in the year 2099, students often stated that they would fit a regression model between water temperature and year, and estimate future water temperatures based on simple linear extrapolation. After completing the “Climate Change Effects on Lake Temperatures” Macrosystems EDDIE module, students’ post‐module assessments instead emphasized how simulation models could provide a range of future lake temperatures, depending on how different drivers of lake temperature changed. This shift in approach lends itself to a more nuanced and realistic understanding of complex and nonlinear relationships in ecology. Moreover, ecology curricula tend to highlight the context‐dependency of ecological phenomena, which can lead students to assume that ecology lacks generalizable patterns. By analyzing high‐frequency and long‐term data from diverse ecosystems, undergraduate students can use their programming skills to test for the generality of patterns across spatial and temporal scales. In addition, learning how to use and write programming scripts empowers students to ask specific ecological questions of their own design, allowing them to move from conducting a suite of predeveloped analyses and visualizations to having complete flexibility and independence to explore data on their own (Baker, 2017), as we observed in several classrooms.

Importantly, the use of EDDIE modules and similar active‐learning tools may aid retention of underrepresented students in STEM fields. Hands‐on, inquiry‐based teaching activities connected to real‐world tasks, such as programming, have been shown to reduce the performance gap between underrepresented minority (URM) students and their non‐URM peers in STEM classes, which has implications for long‐term retention in STEM fields (Alper, 1993; Ballen et al., 2017). Confidence in one's skills and abilities is an important aspect of student retention in computer science and other STEM fields, with a disproportionate effect on women and other underrepresented groups (Anderson, McKenzie, Wellman, Brown, & Vrbsky, 2011). In computer science classrooms, the use of modules to introduce programming topics helps reduce intimidation and increase interest in course content, particularly for students that previously rated themselves as having “average” level skills (Anderson et al., 2011).

For ecology students using EDDIE modules, we similarly found support to Klug et al. (2017) that “those with the most to gain, gain the most,” where post‐module growth in perceived proficiency and confidence in the use of computational tools was highest in students who had the lowest initial scores (Figure 1). This result suggests that using the modules can help equalize student abilities and computational literacy while simultaneously building confidence for all students. Moreover, we expect that these gains contribute to an increased likelihood to continue to pursue computational tools, as has been seen previously in computer science classes (Anderson et al., 2011). For example, a student who self‐reported having “very limited computer modeling experience prior to this activity” reflected that they were more likely to use computer programming after completing a module, as their “first time truly modeling an ecosystem” increased their confidence and proficiency. Thus while the magnitude of growth in self‐assessed proficiency, confidence, and likely future use for any given student after the completion of one module may be small, qualitative student feedback suggests that a single 3‐hour activity can set the stage for ongoing learning and further gains.

Finally, increased computational literacy training will prepare undergraduate students for many fields beyond ecology. Regardless of their future careers, ecology undergraduates will likely need to manipulate and visualize large, heterogeneous datasets, which are now ubiquitous in government, industry, and academia (Mellody, 2014). Increased data science, systems thinking, and quantitative skills are seen as increasingly essential in the workforce (Valle & Berdanier, 2012), and increased computational literacy and programming skills will prepare current students for diverse 21^st^ century careers. Consequently, teaching ecology undergraduates computational literacy will not only advance ecology as a discipline, but will also help build an informed workforce and electorate that is prepared to work with and interpret a wide range of big data.

## CONFLICT OF INTEREST

None declared.

## AUTHOR CONTRIBUTIONS

KJF and CCC conceived the study, analyzed and interpreted the data, and wrote the manuscript.

## DATA ACCESSIBILITY

The data presented in this manuscript are human subjects data, and per our Institutional Review Board permissions, cannot be publicly archived.

## APPENDIX 1 Methods

### 1.1

We compared undergraduate student responses to questions about their proficiency, confidence, and likely future use of different computational tools (Microsoft Excel software, R software, computer programming) before and after their completion of the Project EDDIE “Lake Modeling” or Macrosystems EDDIE “Climate Change Effects on Lake Temperatures” or “Cross‐Scale Emergence” modules (for detailed module information, see Carey & Gougis, 2017). The modules were taught in one introductory (freshman‐level) environmental science course and eight upper‐level (junior and senior‐level) freshwater ecology courses. The introductory course was taught at an associate's college and the eight other courses were taught at four doctorate‐granting universities (R1 and R2) and two master's universities (M1).

For each assessment question, students responded using a Likert scale from 1 (low) to 5 (high; Table A1 for questions). Pre‐module assessments were completed within 10 days prior to the module being taught as part of classroom lecture or lab activities, while post‐module assessments were completed between 1 and 14 days after the module was taught. All assessments were completed online. Only student participants who voluntarily consented to the use of their data were included in our comparison in accordance with Institutional Review Board permissions (Virginia Tech IRB #13‐804; Carleton College IRB #0002470).

**Table A1:** Likert scale for student pre‐ and post‐module self‐assessments. Assessment questions for each computational tool were phrased as, “Using the scale below, how would you rank your [metric] with each of the following computational tools?”

Metric	1	2	3	4	5
Proficiency	No proficiency, not able to apply this tool to an assignment	Basic proficiency, able to handle simple applications of this tool to an assignment	Intermediate proficiency, able to apply this tool independently to many types of assignments	Advanced proficiency, able to apply this tool independently to nearly all types of assignments	Expert proficiency, able to apply this tool independently to all types of assignments and serve as a role model or coach others
Confidence	Not at all confident	Somewhat confident	Moderately confident	Very confident	Completely confident
Likelihood of future use	Extremely unlikely	Unlikely	Neutral	Likely	Extremely likely

We pooled data across all classes due to low sample sizes within each class (*n *=* *7, 10, 12, 13, 14, 21, 22, 24, 24). We tested for changes in student perceptions of their proficiency, confidence, and likely future use of each computational tool (Microsoft Excel, R, computer programming) using Wilcoxon signed‐rank tests of paired student responses from pre‐ and post‐module self‐assessments. *p*‐values were estimated using a normal approximation, with statistical significance being interpreted at *α* = 0.05. We calculated per‐student changes in each metric (proficiency, confidence, likely future use) for each computational tool as the difference between post‐ and pre‐assessment responses. We then tested whether changes (Post – Pre) were correlated with pre‐assessment responses using Spearman's rho correlation coefficients. *p*‐values were estimated using the asymptotic *t* approximation. Effect sizes were calculated as Z/√*n*. All analyses were conducted using R 3.5.0 (R Core Team 2018).

## References

[ece34363-bib-0001] Alper, J. (1993). The pipeline is leaking women all the way along. Science, 260, 409–411. 10.1126/science.260.5106.409 17838262

[ece34363-bib-0002] Anderson, M. , McKenzie, A. , Wellman, B. , Brown, M. , & Vrbsky, S. (2011). Affecting attitudes in first‐year computer science using syntax‐free robotics programming. ACM Inroads, 2, 51–57. 10.1145/2003616.2003635

[ece34363-bib-0003] Baker, M. (2017). Code alert. Nature, 541, 563–565. 10.1038/nj7638-563a

[ece34363-bib-0004] Ballen, C. J. , Wieman, C. , Salehi, S. , Searle, J. B. , & Zamudio, K. R. (2017). Enhancing diversity in undergraduate science: Self‐efficacy drives performance gains with active learning. Cell Biology Education, 16, ar56. 10.1187/cbe.16-12-0344 PMC5749958 29054921

[ece34363-bib-0005] Bennett, S. , & Maton, K. (2010). Beyond the “digital natives” debate: Towards a more nuanced understanding of students’ technology experiences. Journal of Computer Assisted Learning, 26, 321–331. 10.1111/j.1365-2729.2010.00360.x

[ece34363-bib-0006] Blum, J. (2012). Are we breeding a generation of app‐loving, web‐addicted digital illiterates?. Toronto: The Globe and Mail.

[ece34363-bib-0007] Bybee, R. W. , Taylor, J. A. , Gardner, A. , Van, P. , Powell, J. C. , Westbrook, A. , … Knapp, N. (2006). The BSCS 5E instructional model: Origins and effectiveness. A report prepared for the office of science education and national institutes of health.

[ece34363-bib-0008] Carey, C. C. , Gougis, R. D. , Klug, J. L. , O'Reilly, C. M. , & Richardson, D. C. (2015). A model for using environmental data‐driven inquiry and exploration to teach limnology to undergraduates. Limnology and Oceanography Bulletin, 24, 32–35. 10.1002/lob.10020

[ece34363-bib-0009] Carey, C. C. , & Gougis, R. D. (2017). Simulation modeling of lakes in undergraduate and graduate classrooms increases comprehension of climate change concepts and experience with computational tools. Journal of Science Education and Technology, 26, 1–11. 10.1007/s10956-016-9644-2

[ece34363-bib-0010] Durden, J. M. , Luo, J. Y. , Alexander, H. , Flanagan, A. M. , & Grossmann, L. (2017). Integrating “big data” into aquatic ecology: Challenges and opportunities. Limnology and Oceanography Bulletin, 26, 101–108. 10.1002/lob.10213

[ece34363-bib-0011] Elliot A. J., Dweck C. S., & Yeager D. S. (Eds.) (2017). Handbook of competence and motivation: Theory and application, 2nd ed. New York: Guilford Publications.

[ece34363-bib-0012] Feinstein, N. W. , Allen, S. , & Jenkins, E. (2013). Outside the pipeline: Reimagining science education for nonscientists. Science, 340, 314–317. 10.1126/science.1230855 23599483

[ece34363-bib-0013] Hampton, S. , Anderson, S. , Bagby, S. , Gries, C. , Han, X. , Hart, E. , … Zimmerman, N. (2015). The Tao of open science for ecology. Ecosphere, 6, 1–13.

[ece34363-bib-0014] Hampton, S. E. , Strasser, C. A. , Tewksbury, J. J. , Gram, W. K. , Budden, A. E. , Batcheller, A. L. , … Porter, J. H. (2013). Big data and the future of ecology. Frontiers in Ecology and the Environment, 11, 156–162. 10.1890/120103

[ece34363-bib-0015] von Hardenberg, A. , Obeng, A. , Pawlik, A. , Pletzer, A. , Shiklomanov, A. , Fouilloux, A. , … Lapp, Z (2018). Data Carpentry: R for data analysis and visualization of Ecological Data

[ece34363-bib-0016] Heffernan, J. B. , Soranno, P. A. , Angilletta, M. J. , Buckley, L. B. , Gruner, D. S. , Keitt, T. H. , … Weathers, K. C. (2014). Macrosystems ecology: Understanding ecological patterns and processes at continental scales. Frontiers in Ecology and the Environment, 12, 5–14. 10.1890/130017

[ece34363-bib-0017] Klug, J. L. , Carey, C. C. , Richardson, D. C. , & Gougis, R. D. (2017). Analysis of high‐frequency and long‐term data in undergraduate ecology classes improves quantitative literacy. Ecosphere, 8, e01733 10.1002/ecs2.1733

[ece34363-bib-0018] Lawson, B. , Szajda, D. , & Barnett, L. (2013). Introducing computer science in an integrated science course. pp. 128–135. Proceedings of the 44th ACM Technical Symposium. Denver, CO.

[ece34363-bib-0019] Louca, L. T. , & Zacharia, Z. C. (2012). Modeling‐based learning in science education: Cognitive, metacognitive, social, material and epistemological contributions. Educational Review, 64, 471–492. 10.1080/00131911.2011.628748

[ece34363-bib-0020] Mellody, M. (2014). Training students to extract value from big data: Summary of a Workshop. Washington, DC: National Academies Press. 26065052

[ece34363-bib-0021] Michener, W. K. , & Jones, M. B. (2012). Ecoinformatics: Supporting ecology as a data‐intensive science. Trends in Ecology and Evolution, 27, 88–93. 10.1016/j.tree.2011.11.016 22240191

[ece34363-bib-0022] National Research Council (2009). A new biology for the 21st century. Washington, DC: National Academies Press.

[ece34363-bib-0023] O'Reilly, C. M. , Gougis, R. D. , Klug, J. L. , Carey, C. C. , Richardson, D. C. , Bader, N. E. , … Hunter, W. (2017). Using large data sets for open‐ended inquiry in undergraduate science classrooms. BioScience, 67, 1052–1061. 10.1093/biosci/bix118

[ece34363-bib-0024] Prensky, M. (2001). Digital natives, digital immigrants part 1. On the Horizon, 9, 1–6.

[ece34363-bib-0025] R Core Team (2018). R: A language and environment for statistical computing. Vienna, Austria: R Foundation for Statistical Computing.

[ece34363-bib-0026] Read, E. K. , O'Rourke, M. , Hong, G. S. , Hanson, P. C. , Winslow, L. A. , Crowley, S. , … Weathers, K. C. (2016). Building the team for team science. Ecosphere, 7, 1–9.

[ece34363-bib-0027] Schwarz, C. V. , & White, B. Y. (2015). Metamodeling knowledge : Developing students ‘ understanding of scientific modeling. Cognition and Instruction, 23, 165–205.

[ece34363-bib-0028] Sherin, B. (2011). Computational literacy: As essential as ABC? In Sherman M., Inquiry: The School of Education and Social Policy, Northwestern University. http://www.sesp.northwestern.edu/news-center/inquiry/2011-spring/computational-literacy.html. Accessed 20 Nov. 2017.

[ece34363-bib-0029] Sins, P. H. M. , Savelsbergh, E. R. , & van Joolingen, W. R. (2005). The difficult process of scientific modelling: An analysis of novices’ reasoning during computer‐based modelling. International Journal of Science Education, 27, 1695–1721. 10.1080/09500690500206408

[ece34363-bib-0030] Valle, D. , & Berdanier, A. (2012). Computer programming skills for environmental sciences. The Bulletin of the Ecological Society of America, 93, 373–389. 10.1890/0012-9623-93.4.373

[ece34363-bib-0031] Wang, Q. , Myers, M. D. , & Sundaram, D. (2013). Digital natives and digital immigrants: Towards a model of digital fluency. Business and Information Systems Engineering, 5, 409–419. 10.1007/s12599-013-0296-y

[ece34363-bib-0032] Weathers, K. , Hanson, P. C. , Arzberger, P. , Brentrup, J. , Brookes, J. , Carey, C. C. , … Zhu, G. (2013). The Global Lake Ecological Observatory Network (GLEON): The evolution of grassroots network science. Limnology and Oceanography Bulletin, 22, 71–73. 10.1002/lob.201322371

[ece34363-bib-0033] Wilensky, U. , Brady, C. E. , & Horn, M. S. (2014). Fostering computational literacy in science classrooms. Communications of the ACM, 57, 24–28. 10.1145/2633031

[ece34363-bib-0034] Wright, A. , Provost, J. , Roecklein‐Canfield, J. A. , & Bell, E. (2013). Essential concepts and underlying theories from physics, chemistry, and mathematics for “biochemistry and molecular biology” majors. Biochemistry and Molecular Biology Education, 41, 302–308. 24019240 10.1002/bmb.20728

